# Prof. Storer's Lectures

**Published:** 1868-02

**Authors:** 


*Prof. Storer's Lectures.*—We call attention to the advertisement of Prof. Horatio R. Storer of Boston, who delivers his third private course of twelve lectures, at his rooms in that city, during the first fortnight in June. No man is better able to deliver an instructive and interesting series of lectures than Dr. Storer, and we hope that his efforts to cultivate a more intimate knowledge of this important speciality of medicine will be appreciated by the profession, and rewarded by the presence of a large class.

